# High-quality permanent draft genome sequence of *Bradyrhizobium* sp. strain WSM1743 - an effective microsymbiont of an *Indigofera* sp. growing in Australia

**DOI:** 10.1186/s40793-015-0073-2

**Published:** 2015-10-26

**Authors:** Leila Eshraghi, Sofie E. De Meyer, Rui Tian, Rekha Seshadri, Natalia Ivanova, Amrita Pati, Victor Markowitz, Tanja Woyke, Nikos C. Kyrpides, Ravi Tiwari, Ron Yates, John Howieson, Wayne Reeve

**Affiliations:** Centre for Rhizobium Studies, Murdoch University, Murdoch, Western Australia; Centre for Phytophthora Science and Management (CPSM), Murdoch University, Murdoch, Western Australia; DOE Join Genome Institute, Walnut Creek, CA USA; Biological Data Management and Technology Center, Lawrence Berkeley National Laboratory, Berkeley, CA USA; Department of Biological Sciences, King Abdulaziz, Jeddah, Saudia Arabia; Department of Agriculture and Food, South Perth, Western Australia

**Keywords:** Root-nodule bacteria, Nitrogen fixation, Rhizobia, *Alphaproteobacteria*, GEBA-RNB

## Abstract

*Bradyrhizobium* sp. strain WSM1743 is an aerobic, motile, Gram-negative, non-spore-forming rod that can exist as a soil saprophyte or as a legume microsymbiont of an *Indigofera* sp. WSM1743 was isolated from a nodule recovered from the roots of an *Indigofera* sp. growing 20 km north of Carnarvon in Australia. It is slow growing, tolerates up to 1 % NaCl and is capable of growth at 37 °C. Here we describe the features of *Bradyrhizobium* sp. strain WSM1743, together with genome sequence information and its annotation. The 8,341,956 bp high-quality permanent draft genome is arranged into 163 scaffolds and 167 contigs, contains 7908 protein-coding genes and 75 RNA-only encoding genes and was sequenced as part of the Root Nodule Bacteria chapter of the Genomic Encyclopedia of Bacteria and *Archaea* project.

**Electronic supplementary material:**

The online version of this article (doi:10.1186/s40793-015-0073-2) contains supplementary material, which is available to authorized users.

## Introduction

Rhizobia are soil-dwelling bacteria that have acquired the ability to establish associations with leguminous plants to symbiotically fix nitrogen. After infection of the plant, the rhizobia become established within root nodules and can fix atmospheric dinitrogen gas into ammonia using a reaction that is catalyzed by the nitrogenase enzyme [1]. The export of fixed nitrogen to the plant improves growth and productivity under N-limiting environmental conditions. The effective use of the symbiosis leads to sustainable cropping systems with a net positive impact on the environment [2]. In Australia, the majority of productive legumes and their rhizobia in agricultural systems have been deliberately, or accidentally, introduced since European settlement [3]. However, recently, there has been an interest in the diversity of Australian native legumes and their microsymbionts [4].

The northwest of Western Australia is an ideal landscape to discover rhizobia nodulating indigenous legume flora [4] and is an area low in introduced legumes and inoculants. In 1996, an extensive survey was conducted of the area revealing a range of indigenous legume genera including a number of *Indigofera* spp. [4]. In Australia, this species has been found at dispersed locations in the Northern Territory, Queensland and Western Australia on dark brown clay loams and frequently on lands under cultivation [5]. The Australian *Indigofera* spp., based on their habitat, can be placed into three categories; i) shrubs, including *I. brevidens*, *I. australis*, *I. adesmiifolia* and some members of the *I. pratensis* group, which occur mainly on better soil types in the east coast, ii) perennial herbs, such as *I. baileyi*, *I. efoliata*, *I. triflora*, *I. georgei*, *I. rugosa* and members of the *I. triflora* and *I. pratensis* groups, which occur in the more arid, or seasonally dry, parts of Australia, iii) annual herbs, uncommon amongst the endemic species, including the two annual species, *I. haplophylla* and *I. ammobia*, which occur in the monsoon tropics and the Tanami and Great Sandy Deserts, respectively [6]. The native species with wide extra-Australian distributions (particularly *I. colutea*, *I. hirsuta*, *I. linnaei* and *I. linifolia*) occur in a variety of habitats, mostly towards the northern parts of Australia. It is likely that these taxa now inhabit a greater range than they did before European settlement, and the Australian populations of these species may have been augmented by the introduction of seed from non-Australian sources [6].

Since there is a paucity of information regarding microsymbionts of *Indigofera*, a collection of root nodules was therefore obtained from the most prevalent *Indigofera* spp. present in northwest Australia and the microsymbionts from these nodules were then isolated. One microsymbiont, *Bradyrhizobium* sp. strain WSM1743, was isolated from a nodule recovered from the roots from an indigenous *Indigofera* sp. growing in red-brown sandy loam 40 m above sea level. The plant was located in natural bush land, approximately 20 km Northeast of the town Carnarvon in Western Australia [4]. The collection area has a warm semi-arid climate with a long-term mean seasonal rainfall of 226 mm.

Strain WSM1743 was identified as a *Bradyrhizobium* sp. based on 16S rRNA typing [4]. Most *Bradyrhizobium* spp., including WSM1743, cannot grow on sucrose or lactose, which may indicate the lack of a disaccharide uptake system [7]. However, WSM1743, unlike other *Bradyrhizobium* spp., is able to grow at 37 °C and this ability could be a specific adaptation to the high soil temperatures experienced in the northwest of Western Australia [4]. Here we present a summary classification and a set of general features for this microsymbiont together with a description of its genome sequence and annotation done as part of the DOE Joint Genome Institute 2010 Genomic Encyclopedia for Bacteria and Archaea-Root Nodule Bacteria project [8] (Additional file 2).

### Organism information

#### Classification and features

**Fig. 1:**
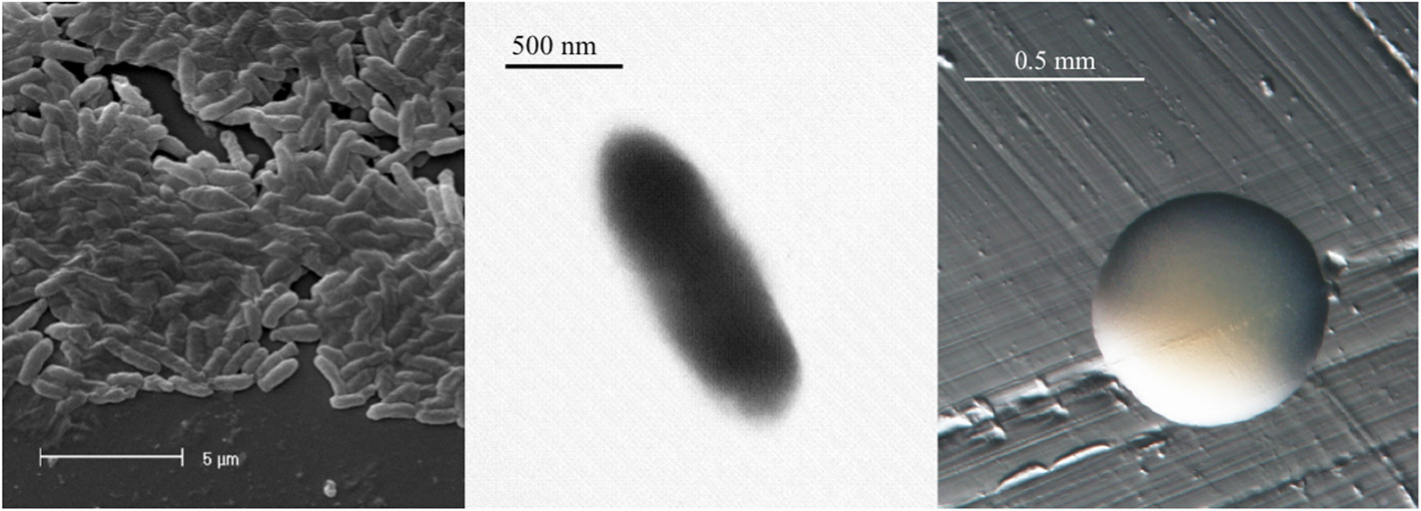
Images of *Bradyrhizobium* sp. strain WSM1743 using scanning (*Left*) and transmission (*Center*) electron microscopy as well as light microscopy to visualize the colony morphology on a solid media (*Right*)

*Bradyrhizobium* sp. strain WSM1743 is a motile, Gram-negative non-spore-forming rod (Fig. 1 Left and Center) in the order *Rhizobiales* of the class *Alphaproteobacteria*. It is slow growing, forming colonies within 7–10 days when grown on half strength Lupin Agar [9] at 28 °C. Colonies on ½LA are white-opaque, slightly domed and moderately mucoid with smooth margins (Fig. 1 Right). This strain was isolated together with 7 other bacteria from native *Indigofera* plants and physiologically characterised. Strain WSM1743 was identified as slow growing with poor growth in 1 % NaCl, no growth at 2 to 3 % NaCl and average growth in pH5 to 9 [4]. *Bradyrhizobium* type strains have a slow generation time (9 to 18 h) and fail to grow in media containing 2 % NaCl [10], which indicates that WSM1743 belongs to this genus. The maximal growth temperature for most *Bradyrhizobium* strains is 33 to 35 °C, with many strains failing to grow above 34 °C [10]. However, WSM1743 was able to grow at 37 °C on ½LA medium [4], and therefore extending the temperature range for *Bradyrhizobium*.

**Fig. 2:**
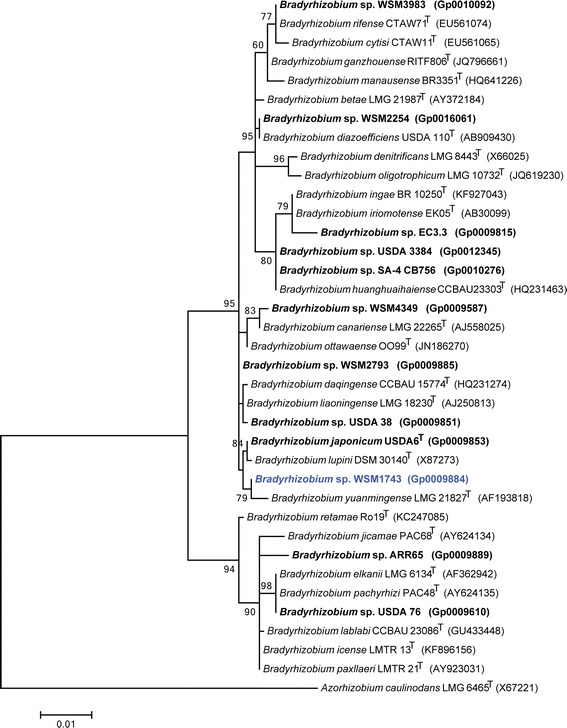
Phylogenetic tree highlighting the position of *Bradyrhizobium* sp. strain WSM1743 (shown in blue print) relative to other type and non-type strains in the *Bradyrhizobium* genus using a 1,251 bp internal region of the 16S rRNA gene. *Azorhizobium caulinodans* LMG 6465^T^ sequence was used as an outgroup. All sites were informative and there were no gap-containing sites. Phylogenetic analyses were performed using MEGA, version 5.05 [17]. The tree was built using the maximum likelihood method with the General Time Reversible model. Bootstrap analysis with 500 replicates was performed to assess the support of the clusters. Type strains are indicated with a superscript T. Strains with a genome sequencing project registered in GOLD [18] are shown in bold and have the GOLD ID mentioned after the strain number, otherwise the NCBI accession number is provided

Figure 2 shows the phylogenetic relationship of *Bradyrhizobium* sp. strain WSM1743 in a 16S rRNA gene sequence based tree. This strain is phylogenetically the most related to the RNB type strains *B. japonicum*USDA 6^T^, *B. lupini*DSM30140^T^ and *B. yuanmingense*LMG 21827^T^ with sequence identities to the WSM1743 16S rRNA gene sequence of 99.78 %, 99.71 % and 99.63 %, respectively, as determined using the EzTaxon-e server [11]. *B. japonicum*USDA6^T^ was originally isolated in Japan from *Glycine max* root nodules and is able to nodulate and fix nitrogen effectively with several other *Glycine* species and *Macroptillium atropurpureum* [12]. *B. lupini*DSM30140^T^ is a microsymbiont of *Lupinus luteus* and *Lupinus angustifolius* [13]. *B. yuanmingense* B071^T^ was isolated from *Lespedeza cuneata* root nodules from China but is also able to nodulate and fix nitrogen effectively with *Vigna unguiculata* and *Glycyrrhiza uralensis* [14]. Additionally, a recent report showed that *B. yuanmingense* and *B. japonicum* are the preferred microsymbionts of *Vigna unguiculata* and *Vigna radiate* in the subtropical region of China [15].

Minimum Information about the Genome Sequence (MIGS) [16] of WSM1743 is provided in Table 1 and Additional file 1: Table S1.

**Table 1:** Classification and general features of *Bradyrhizobium* sp. strain WSM1743 in accordance with the MIGS recommendations [15] published by the Genome Standards Consortium [19]

MIGS ID	Property	Term	Evidence code
	Current classification	Domain *Bacteria*	TAS [20]
		Phylum *Proteobacteria*	TAS [21, 22]
		Class *Alphaproteobacteria*	TAS [21]
		Order *Rhizobiales*	TAS [23]
		Family *Bradyrhizobiaceae*	TAS [24]
		Genus *Bradyrhizobium*	TAS [10]
		Species sp.	IDA
	Gram stain	Negative	TAS [12]
	Cell shape	Rod	IDA
	Motility	Motile	IDA
	Sporulation	Non-sporulating	IDA
	Temperature range	Mesophile	IDA
	Optimum temperature	28 °C	IDA
	pH range; Optimum	5 - 9	TAS [4]
	Carbon source	Glutamate, L-arabinose	TAS [4]
MIGS-6	Habitat	Soil, root nodule, on host	IDA
MIGS-6.3	Salinity	Not reported	
MIGS-22	Oxygen requirement	Aerobic	IDA
MIGS-15	Biotic relationship	Free living, symbiotic	IDA
MIGS-14	Pathogenicity	Non-pathogenic	NAS
MIGS-4	Geographic location	20 km north of Carnarvon	TAS [4]
MIGS-5	Nodule collection date	July 1996	TAS [4]
MIGS-4.1	Latitude	−24.770	IDA
MIGS-4.2	Longitude	113.702	IDA
MIGS-4.3	Depth	Up to 1 m	IDA
MIGS-4.4	Altitude	11 m	IDA

Evidence codes – *IDA* Inferred from Direct Assay; *TAS* Traceable Author Statement (i.e., a direct report exists in the literature); *NAS* Non-traceable Author Statement (i.e., not directly observed for the living, isolated sample, but based on a generally accepted property for the species, or anecdotal evidence). These evidence codes are from the Gene Ontology project [25]

### Symbiotaxonomy

*Bradyrhizobium* sp. strain WSM1743 was isolated from *Indigofera* sp. nodules collected at site 32, north of Carnarvon, Western Australia [4]. The site of collection contained several Australian native legumes, with a soil pH of 7.5. Symbiotic interactions of *Bradyrhizobium* sp. strain WSM1743 were assessed on three annual, one biennial and nine perennial exotic legume species that have agricultural use, or potential use, in southern Australia. WSM1743 consistently nodulated with the exotic legume species, *Macroptilium atropurpureum* and *Phaseolus vulgaris*, inconsistently with *Ononis natrix* and did not form nodules with *Argyrolobium uniflorum**, Chamaecytisus proliferus,**Sutherlandia microphylla**,**Hedysarum coronarium**,**Medicago sativa**,**Ornithopus sativus**, O. compressus,**Trifolium burchellianum**, T. polymorphum* and *T. uniflorum*. Strain WSM1743 was able to consistently nodulate the Australian native legumes, *Acacia saligna**,**Kennedia prorepens* and *K. coccinea*, but could not nodulate *Swainsona pterostylis**, S. formosa* and *S. macculochiana* [4]. Additionally it was noted that the isolate could not nodulate *Indigofera brevidens*, an indigenous *Indigofera* found in the same location as the host of WSM1743 [4].

## Genome sequencing information

### Genome project history

This organism was selected for sequencing on the basis of its environmental and agricultural relevance to issues in global carbon cycling, alternative energy production, and biogeochemical importance, and is part of the Genomic Encyclopedia of Bacteria and *Archaea*, The Root Nodulating Bacteria chapter (GEBA-RNB) project at the U.S. Department of Energy, Joint Genome Institute [8]. The genome project is deposited in the Genomes OnLine Database [18] and the high-quality permanent draft genome sequence in IMG [26]. Sequencing, finishing and annotation were performed by the JGI using state of the art sequencing technology [27]. A summary of the project information is shown in Table 2.

**Table 2:** Genome sequencing project information for *Bradyrhizobium* sp. strain WSM1743

MIGS ID	Property	Term
MIGS-31	Finishing quality	High-quality permanent draft
MIGS-28	Libraries used	Illumina Std PE
MIGS-29	Sequencing platforms	Illumina Hiseq 2000
MIGS-31.2	Fold coverage	440× Illumina
MIGS-30	Assemblers	Velvet 1.1.04; ALLPATHS-LG V. r39750
MIGS-32	Gene calling method	Prodigal 1.4
	Locus Tag	YU9
	Genebank ID	AXAZ00000000
	Genbank date of release	December 12, 2013
	GOLD ID	Gp0009884 [28]
	BIOPROJECT	PRJNA162991
MIGS-13	Source material identifier	WSM1743
	Project relevance	Symbiotic N_2_ fixation, agriculture

### Growth conditions and genomic DNA preparation

*Bradyrhizobium* sp. strain WSM1743 was cultured to mid logarithmic phase in 60 ml of TY rich media on a gyratory shaker at 28 °C [29]. DNA was isolated from the cells using a Cetyl trimethyl ammonium bromide bacterial genomic DNA isolation method [30].

### Genome sequencing and assembly

The draft genome of *Bradyrhizobium* sp. strain WSM1743 was generated at the DOE Joint Genome Institute [31]. An Illumina standard shotgun library was constructed and sequenced using the Illumina HiSeq 2000 platform, which generated 14,683,452 reads totaling 2.2 Gbp. All general aspects of library construction and sequencing performed at the JGI can be found at the JGI web site [31]. All raw Illumina sequence data was passed through DUK, a filtering program developed at JGI, which removes known Illumina sequencing and library preparation artifacts (Mingkun L, Copeland A, Han J. unpublished). Artifact filtered sequence data was then screened and trimmed according to the k–mers present in the dataset (Mingkun L, Copeland A, Han J. unpublished). High–depth k–mers, presumably derived from MDA amplification bias, cause problems in the assembly, especially if the k–mer depth varies in orders of magnitude for different regions of the genome. Reads with high k–mer coverage (>30x average k–mer depth) were normalized to an average depth of 30x. Reads with an average kmer depth of less than 2x were removed. Following steps were then performed for assembly: (1) normalized Illumina reads were assembled using Velvet version 1.1.04 [32] (2) 1–3 Kbp simulated paired end reads were created from Velvet contigs using wgsim [33] (3) normalized Illumina reads were assembled with simulated read pairs using Allpaths–LG (version r39750) [34]. Parameters for assembly steps were: 1) Velvet optimizing parameters (−−v --s 51 --e 71 --i 2 --t 1 --f "-shortPaired -fastq $FASTQ" --o "-ins_length 250 -min_contig_lgth 500) 2) wgsim version 0.3.0 (−e 0–1 76–2 76 -r 0 -R 0 -X 0) 3) Allpaths–LG (PrepareAllpathsInputs: PHRED 64 = 1 PLOIDY = 1 FRAG COVERAGE = 125 JUMP COVERAGE = 25 LONG JUMP COV = 50, RunAllpathsLG: THREADS = 8 RUN = std_shredpairs TARGETS = standard VAPI_WARN_ONLY = True OVERWRITE = True). The final draft assembly contained 167 contigs in 163 scaffolds. The total size of the genome is 8.3 Mbp and the final assembly is based on 1238 Mbp of Illumina data, which provides an average of 440x coverage.

### Genome annotation

Genes were identified using Prodigal [35], as part of the DOE-JGI genome annotation pipeline [36, 37]. The predicted CDSs were translated and used to search the National Center for Biotechnology Information non-redundant database, UniProt, TIGRFam, Pfam, KEGG, COG, and InterPro databases. The tRNAScanSE tool [38] was used to find tRNA genes, whereas ribosomal RNA genes were found by searches against models of the ribosomal RNA genes built from SILVA [39]. Other non–coding RNAs such as the RNA components of the protein secretion complex and the RNase P were identified by searching the genome for the corresponding Rfam profiles using INFERNAL [40]. Additional gene prediction analysis and manual functional annotation was performed within the Integrated Microbial Genomes-Expert Review system [41] developed by the Joint Genome Institute, Walnut Creek, CA, USA.

## Genome properties

The genome is 8,341,956 nucleotides with 63.37 % GC content (Table 3) and comprised of 163 scaffolds of 167 contigs. From a total of 7983 genes, 7908 were protein encoding and 75 RNA only encoding genes. The majority of genes (71.51 %) were assigned a putative function whilst the remaining genes were annotated as hypothetical. The distribution of genes into COG functional categories is presented in Table 4.

**Table 3:** Genome statistics for *Bradyrhizobium* sp. strain WSM1743

Attribute	Value	% of total
Genome size (bp)	8,341,956	100.00
DNA coding (bp)	6,951,810	83.34
DNA G + C (bp)	5,286,166	63.37
DNA scaffolds	163	
Total genes	7,983	100.00
Protein-coding genes	7,908	99.06
RNA genes	75	0.94
Pseudo genes	12	0.15
Genes in internal clusters	465	5.82
Genes with function prediction	5,709	71.51
Genes assigned to COGs	4,824	60.43
Genes with Pfam domains	6,012	75.31
Genes with signal peptides	840	10.52
Genes with transmembrane proteins	1,784	22.35
CRISPR repeats	1

**Table 4:** Number of protein coding genes of *Bradyrhizobium* sp. strain WSM1743 associated with the general COG functional categories

Code	Value	% age	Description
J	190	3.51	Translation, ribosomal structure and biogenesis
A	0	0.00	RNA processing and modification
K	369	6.82	Transcription
L	155	2.86	Replication, recombination and repair
B	2	0.04	Chromatin structure and dynamics
D	28	0.52	Cell cycle control, Cell division, chromosome partitioning
V	82	1.51	Defense mechanisms
T	213	3.93	Signal transduction mechanisms
M	253	4.67	Cell wall/membrane/envelope biogenesis
N	94	1.74	Cell motility
U	112	2.07	Intracellular trafficking, secretion, and vesicular transport
O	180	3.33	Posttranslational modification, protein turnover, chaperones
C	357	6.60	Energy production and conversion
G	403	7.45	Carbohydrate transport and metabolism
E	642	11.86	Amino acid transport and metabolism
F	88	1.63	Nucleotide transport and metabolism
H	207	3.82	Coenzyme transport and metabolism
I	333	6.15	Lipid transport and metabolism
P	285	5.27	Inorganic ion transport and metabolism
Q	242	4.47	Secondary metabolite biosynthesis, transport and catabolism
R	659	12.17	General function prediction only
S	519	9.59	Function unknown
-	3,159	39.57	Not in COGS

The total is based on the total number of protein coding genes in the genome

## Conclusion

*Bradyrhizobium* sp. WSM1743 belongs to a group of Alpha-rhizobia microsymbionts from native Australian legumes and was isolated from a nodule of an *Indigofera* species growing 20 km north of Carnarvon in northwestern Australia. Phylogenetic analysis revealed that WSM1743 is most closely related to *B. japonicum*USDA 6^T^, which was obtained from *Glycine max* root nodules from Japan and is able to nodulate and fix nitrogen effectively with several other *Glycine* species and *Macroptillium atropurpureum* [12]. Strain WSM1743 has been shown to nodulate with *Macroptilium atropurpureum* and endemic Australian legumes including *Acacia saligna*, *Kennedia prorepens* and *K. coccinea* [4].

A comparison of the genome of WSM1743 to that of USDA 6^T^ [42] reveals that WSM1743 has a lower GC content, gene count, coding base count %, rRNAcount, COG % and transmembrane %. In contrast, the paralogs % is much higher for WSM1743 than for USDA 6^T^ (81.75 % versus 42.54 %). These two genomes are included within a group of 54 *Bradyrhizobium* genomes that have been deposited into the IMG database [26]. Within this group, strains known to symbiotically fix nitrogen all contain the nitrogenase-RXN MetaCyc pathway that is characterized by the multiprotein nitrogenase complex. The genome of *Bradyrhizobium* sp. WSM1743, in conjunction with the other *Bradyrhizobium* genomes, will be important for on-going comparative and functional analyses of the plant microbe interactions required for the successful establishment of native Australian legume symbioses.

## Additional files

Additional file 1: Table S1. Associated MIGS record for WSM1743. (PDF 74 kb) Additional file 2:
**Annotation Summary, GenBank Accession Summary, Strain ID Summary, Plant Name Summary, Scientific Name Summary and Reference Search Summary.** (DOC 78 kb)

## Abbreviations

GEBA-RNB: Genomic encyclopedia of bacteria and archaea – root nodule bacteria
JGI: Joint genome institute
TY: Trypton yeast
CTAB: Cetyl trimethyl ammonium bromide
WSM: Western Australian soil microbiology
IMG-ER: Integrated microbial genomes-expert review
NCBI: National centre for biotechnology information
N_2_: Dinitrogen
