# Supplementary material for: High-quality permanent draft genome sequence of Bradyrhizobium sp. strain WSM1743 - an effective microsymbiont of an Indigofera sp. growing in Australia
Source: Stand Genomic Sci. 2015 Oct 26;10:87. doi: 10.1186/s40793-015-0073-2 (PMC4623297; doi:10.1186/s40793-015-0073-2)
Supplement: Additional file 1: Table S1. — Associated MIGS record for WSM1743. (PDF 74 kb) [file 40793_2015_73_MOESM1_ESM.pdf]

Table S1. Associated MIGS record for WSM1743

MIGS-ID	Field name	Description
MIGS-1	Submit to INSDC/Trace archives	
1.1	PID	
1.2	Trace Archive	
MIGS-2	MIGS CHECK LIST TYPE	
MIGS-3	Project Name	GEBA - Root Nodulating Bacteria
MIGS-4	Geographic Location	20 km north of Carnarvon, Australia
4.1	Latitude	-24.770
4.2	Longitude	113.702
4.3	Depth	
4.4	Altitude	
MIGS-5	Time of Sample collection	
MIGS-6	Habitat (EnvO)	Host, Plant root, Root nodule, Soil
6.1	Temperature	28
6.2	pH	7.5
6.3	Salinity	
6.4	Chlorophyll	
6.5	Conductivity	
6.6	Light intensity	
6.7	Dissolved organic carbon (DOC)	
6.8	Current	
6.9	Atmospheric data	
6.10	Density	
6.11	Alkalinity	
6.12	Dissolved oxygen	
6.13	Particulate organic carbon (POC)	
6.14	Phosphate	
6.15	Nitrate	
6.16	Sulfates	
6.17	Sulfides	
6.18	Primary production	
MIGS-7	Subspecific genetic lineage	
MIGS-9	Number of replicons	
MIGS-10	Extrachromosomal elements	
MIGS-11	Estimated Size	8.3 Mbp
MIGS-12	Reference for biomaterial or Genome report	
MIGS-13	Source material identifiers	
MIGS-14	Known Pathogenicity	Non-pathogen
MIGS-15	Biotic Relationship	Symbiotic
MIGS-16	Specific Host	*Indigofera* sp.
MIGS-17	Host specificity or range (taxid)	
MIGS-18	Health status of Host	
MIGS-19	Trophic Level	
MIGS-22	Relationship to Oxygen	Aerobe
MIGS-23	Isolation and Growth conditions	TY media, 28°C, aerobe
MIGS-27	Nucleic acid preparation	CTAB
MIGS-28	Library construction	Illumina Std PE
28.1	Library size	2.2 Mbp
28.2	Number of reads	14,683,452
28.3	Vector	
MIGS-29	Sequencing method	Illumina HiSeq 2000
MIGS-30	Assembly	
30.1	Assembly method	ALLPATHS-LG v. r37654
30.2	Estimated error rate	
30.3	Method of calculation	
MIGS-31	Finishing strategy	
31.1	Status	High-quality permanent draft
31.2	Coverage	440x
31.3	Contigs	167
MIGS-32	Relevant SOPs	
MIGS-33	Relevant e-resources	
