# Supplementary material for: High-quality permanent draft genome sequence of Bradyrhizobium sp. strain WSM1743 - an effective microsymbiont of an Indigofera sp. growing in Australia
Source: Stand Genomic Sci. 2015 Oct 26;10:87. doi: 10.1186/s40793-015-0073-2 (PMC4623297; doi:10.1186/s40793-015-0073-2)
Supplement: Additional file 2: — Annotation Summary, GenBank Accession Summary, Strain ID Summary, Plant Name Summary, Scientific Name Summary and Reference Search Summary. (DOC 78 kb) [file 40793_2015_73_MOESM2_ESM.doc]

**Annotation Summary**

| Name | Count | References | Comments |
| --- | --- | --- | --- |
| Alphaproteobacteria | 2 | CLASS Alphaproteobacteria:  G.M. Garrity, J.A. Bell, T. Lilburn: Class I. Alphaproteobacteria class. nov. Bergey's Manual of Systematic Bacteriology 2005, 2, Part C: 1.  List Editor: Validation List No. 107. List of new names and new combinations previously effectively, but not validly, published. International Journal of Systematic and Evolutionary Microbiology 2006, 56: 1-6. |  |
| Azorhizobium caulinodans | 1 | SPECIES Azorhizobium caulinodans:  B. Dreyfus, J.L. Garcia, M. Gillis: Characterization of Azorhizobium caulinodans gen. nov. a stem nodulating nitrogen-fixing bacterium isolated from Sesbania rastrata. International Journal of Systematic Bacteriology 1988, 38: 89-98. |  |
| Bradyrhizobiaceae | 1 | FAMILY Bradyrhizobiaceae:  List Editor: Validation List No. 107. List of new names and new combinations previously effectively, but not validly, published. International Journal of Systematic and Evolutionary Microbiology 2006, 56: 1-6.  G.M. Garrity, J.A. Bell, T. Lilburn: Family VII. Bradyrhizobiaceae fam. nov. Bergey's Manual of Systematic Bacteriology 2005, 2, Part C: 438. |  |
| Bradyrhizobium | 9 | GENUS Bradyrhizobium:  D.C. Jordan: Transfer of Rhizobium japonicum Buchanan 1980 to Bradyrhizobium gen. nov., a genus of slow-growing, root nodule bacteria from leguminous plants. International Journal of Systematic Bacteriology 1982, 32: 136-139. |  |
| Bradyrhizobium japonicum | 4 | SPECIES Bradyrhizobium japonicum:  D.C. Jordan: Transfer of Rhizobium japonicum Buchanan 1980 to Bradyrhizobium gen. nov., a genus of slow-growing, root nodule bacteria from leguminous plants. International Journal of Systematic Bacteriology 1982, 32: 136-139. |  |
| Bradyrhizobium lupini | 2 | SPECIES Bradyrhizobium lupini:  A. Peix, M.H. Ramírez-Bahena, J.D. Flores-Félix, P.A. de la Vega, R. Rivas, P.F. Mateos, J.M. Igual, E. Martínez-Molina, M.E. Trujillo-Toledo, E. Velázquez: Revision of the taxonomic status of the species Rhizobium lupini and reclassification as Bradyrhizobium lupini comb. nov. International Journal of Systematic and Evolutionary Microbiology 2015, 65: 1213-1219.  SPECIES Bosea lupini:  S.E. De Meyer, A. Willems: Multilocus sequence analysis of Bosea species and description of Bosea lupini sp. nov., Bosea lathyri sp. nov. and Bosea robiniae sp. nov., isolated from legumes. International Journal of Systematic and Evolutionary Microbiology 2012, 62: 2505-2510. | 'B. lupini' resolved to multiple names (homonyms):  <http://dx.doi.org/10.1601/nm.26558>  species Bradyrhizobium lupini  <http://dx.doi.org/10.1601/nm.23419>  species Bosea lupini  Please verify that the appropriate name was chosen and update the link to the appropriate DOI if necessary.  If it has not already been made clear from the context of the name in this document, consider revising to remove ambiguity. |
| Bradyrhizobium yuanmingense | 3 | SPECIES Bradyrhizobium yuanmingense:  Z.Y. Yao, F.L. Kan, E.T. Wang, G.H. Wei, W.X. Chen: Characterization of rhizobia that nodulate legume species of the genus Lespedeza and description of Bradyrhizobium yuanmingense sp. nov. International Journal of Systematic and Evolutionary Microbiology 2002, 52: 2219-2230. |  |
| Proteobacteria | 1 | PHYLUM Proteobacteria:  G.M. Garrity, J.A. Bell, T. Lilburn: Phylum XIV. Proteobacteria phyl. nov. Bergey's Manual of Systematic Bacteriology 2005, 2, Part B: 1. |  |
| Rhizobiales | 2 | ORDER Rhizobiales:  List Editor: Validation List No. 107. List of new names and new combinations previously effectively, but not validly, published. International Journal of Systematic and Evolutionary Microbiology 2006, 56: 1-6.  L.D. Kuykendall: Order VI. Rhizobiales ord. nov. Bergey's Manual of Systematic Bacteriology 2005, 2, Part C: 324. |  |
| Rhizobium | 1 | GENUS Rhizobium:  V.B.D. Skerman, V. McGowan, P.H.A. Sneath: Approved Lists of Bacterial Names. International Journal of Systematic Bacteriology 1980, 30: 225-420.  B. Frank: Über die Pilzsymbiose der Leguminosen. Berichte der Deutschen Botanischen Gesellschaft 1889, 7: 332-346. |  |

**GenBank Accession Summary**

| GenBank Accession | Summary |
| --- | --- |
| PRJNA162991 | BioProject PRJNA162991: http://www.ncbi.nlm.nih.gov/bioproject/PRJNA162991 |
| AXAZ00000000 | AXAZ00000000.1 is a bacterial sequences record containing linear, double-stranded DNA (168 bases) from Bradyrhizobium sp. strain WSM1743. The record was created on September 30, 2013 and last updated December 12, 2013. It contains 1 feature. |

**Strain ID Summary**

| Strain ID | Summary |
| --- | --- |
| LMG 6465T | Collection Code: LMG  Collection Name: Belgian Coordinated Collections of Microorganisms/ LMG Bacteria Collection  Institution: LMG (Belgian Coordinated Collections of Microorganisms/ LMG Bacteria Collection) - Belgium  Strain ID: LMG 6465T |
| WSM1743 | Collection Code: WSM  Collection Name: Weston-super-Mare Museum and Art Gallery  Institution: WSM (Weston-super-Mare Museum and Art Gallery) - The United Kingdom  Strain ID: WSM1743 |
| USDA 6T | Collection Code: USDA  Collection Name: United States Department of Agriculture  Institution: USDA (United States Department of Agriculture) - The United States  Strain ID: USDA 6T |
| USDA6T | Collection Code: USDA  Collection Name: United States Department of Agriculture  Institution: USDA (United States Department of Agriculture) - The United States  Strain ID: USDA6T |
| DSM30140T | Collection Code: DSM  Collection Name: Deutsche Sammlung von Mikroorganismen und Zellkulturen GmbH  Institution: DSM (Deutsche Sammlung von Mikroorganismen und Zellkulturen GmbH) - Deutschland  Strain ID: DSM30140T  External link:  <http://www.dsmz.de/catalogues/details/culture/DSM-30140.html> |
| LMG 21827T | Collection Code: LMG  Collection Name: Belgian Coordinated Collections of Microorganisms/ LMG Bacteria Collection  Institution: LMG (Belgian Coordinated Collections of Microorganisms/ LMG Bacteria Collection) - Belgium  Strain ID: LMG 21827T |

**Plant Name Summary**

| Name | Summary |
| --- | --- |
| Lupinus angustifolius | Kew Taxonomy ID: tro-13036916  Name resolves to multiple resolutions in Kew.  Links:  <http://www.theplantlist.org/tpl1.1/record/tro-13036916>  <http://www.theplantlist.org/tpl1.1/record/ild-8552>  USDA Taxonomy ID: LUAN4  Links:  <http://plants.usda.gov/core/profile?symbol=LUAN4> |
| Glycyrrhiza uralensis | Kew Taxonomy ID: tro-13019879  Name resolves to multiple resolutions in Kew.  Links:  <http://www.theplantlist.org/tpl1.1/record/tro-13019879>  <http://www.theplantlist.org/tpl1.1/record/ild-32406> |
| Glycine max | Kew Taxonomy ID: ild-2760  Links:  <http://www.theplantlist.org/tpl1.1/record/ild-2760>  USDA Taxonomy ID: GLMA4  Links:  <http://plants.usda.gov/core/profile?symbol=GLMA4> |
| Hedysarum coronarium | Kew Taxonomy ID: ild-5100  Links:  <http://www.theplantlist.org/tpl1.1/record/ild-5100>  USDA Taxonomy ID: HECO24  Links:  <http://plants.usda.gov/core/profile?symbol=HECO24> |
| Medicago sativa | Kew Taxonomy ID: ild-8536  Links:  <http://www.theplantlist.org/tpl1.1/record/ild-8536>  USDA Taxonomy ID: MESA  Name resolves to multiple resolutions in USDA.  Links:  <http://plants.usda.gov/core/profile?symbol=MESA>  <http://plants.usda.gov/core/profile?symbol=MESAS>  <http://plants.usda.gov/core/profile?symbol=MESAT> |
| Trifolium burchellianum | Kew Taxonomy ID: ild-8052  Links:  <http://www.theplantlist.org/tpl1.1/record/ild-8052>  USDA Taxonomy ID: TRBU4  Links:  <http://plants.usda.gov/core/profile?symbol=TRBU4> |
| Acacia saligna | Kew Taxonomy ID: tro-13024476  Name resolves to multiple resolutions in Kew.  Links:  <http://www.theplantlist.org/tpl1.1/record/tro-13024476>  <http://www.theplantlist.org/tpl1.1/record/ild-591>  USDA Taxonomy ID: ACSA  Links:  <http://plants.usda.gov/core/profile?symbol=ACSA> |
| Indigofera brevidens | Kew Taxonomy ID: ild-35103  Links:  <http://www.theplantlist.org/tpl1.1/record/ild-35103> |
| Macroptilium atropurpureum | Kew Taxonomy ID: ild-2784  Links:  <http://www.theplantlist.org/tpl1.1/record/ild-2784>  USDA Taxonomy ID: MAAT80  Links:  <http://plants.usda.gov/core/profile?symbol=MAAT80> |
| Indigofera | USDA Taxonomy ID: INDIG  Links:  <http://plants.usda.gov/core/profile?symbol=INDIG> |
| Ononis natrix | Kew Taxonomy ID: ild-7958  Links:  <http://www.theplantlist.org/tpl1.1/record/ild-7958>  USDA Taxonomy ID: ONNA  Links:  <http://plants.usda.gov/core/profile?symbol=ONNA> |
| Sutherlandia microphylla | Kew Taxonomy ID: tro-13033260  Name resolves to multiple resolutions in Kew.  Links:  <http://www.theplantlist.org/tpl1.1/record/tro-13033260>  <http://www.theplantlist.org/tpl1.1/record/ild-7736>  USDA Taxonomy ID: SUMI2  Links:  <http://plants.usda.gov/core/profile?symbol=SUMI2> |
| Argyrolobium uniflorum | Kew Taxonomy ID: ild-8309  Links:  <http://www.theplantlist.org/tpl1.1/record/ild-8309>  USDA Taxonomy ID: ARUN5  Links:  <http://plants.usda.gov/core/profile?symbol=ARUN5> |
| Kennedia prorepens | Kew Taxonomy ID: ild-32183  Links:  <http://www.theplantlist.org/tpl1.1/record/ild-32183> |
| Ornithopus sativus | Kew Taxonomy ID: ild-5208  Links:  <http://www.theplantlist.org/tpl1.1/record/ild-5208>  USDA Taxonomy ID: ORSA2  Name resolves to multiple resolutions in USDA.  Links:  <http://plants.usda.gov/core/profile?symbol=ORSA2>  <http://plants.usda.gov/core/profile?symbol=ORSAS> |
| Lespedeza cuneata | USDA Taxonomy ID: LECU  Links:  <http://plants.usda.gov/core/profile?symbol=LECU> |
| Phaseolus vulgaris | Kew Taxonomy ID: ild-2934  Links:  <http://www.theplantlist.org/tpl1.1/record/ild-2934>  USDA Taxonomy ID: PHVU  Links:  <http://plants.usda.gov/core/profile?symbol=PHVU> |
| Swainsona pterostylis | Kew Taxonomy ID: ild-34773  Links:  <http://www.theplantlist.org/tpl1.1/record/ild-34773> |
| Lupinus luteus | Kew Taxonomy ID: ild-8560  Links:  <http://www.theplantlist.org/tpl1.1/record/ild-8560>  USDA Taxonomy ID: LULU80  Links:  <http://plants.usda.gov/core/profile?symbol=LULU80> |
| Vigna unguiculata | Kew Taxonomy ID: ild-3589  Links:  <http://www.theplantlist.org/tpl1.1/record/ild-3589>  USDA Taxonomy ID: VIUN  Name resolves to multiple resolutions in USDA.  Links:  <http://plants.usda.gov/core/profile?symbol=VIUN>  <http://plants.usda.gov/core/profile?symbol=VIUNU> |

**Scientific Name Summary**

| Name | Summary |
| --- | --- |
| Phytophthora | NCBI Taxonomy ID: 4783  Links:  <http://www.ncbi.nlm.nih.gov/Taxonomy/Browser/wwwtax.cgi?lvl=0&id=4783> |
| Meta | NCBI Taxonomy ID: 94025  Links:  <http://www.ncbi.nlm.nih.gov/Taxonomy/Browser/wwwtax.cgi?lvl=0&id=94025> |
| Indigofera | NCBI Taxonomy ID: 20685  Links:  <http://www.ncbi.nlm.nih.gov/Taxonomy/Browser/wwwtax.cgi?lvl=0&id=20685> |

**Reference Search Summary**

| Name | Occurence |
| --- | --- |
| Stand Genomic Sci | 1 |
| Standards in Genomic Sciences | 1 |
